# Supplementary material for: Primipaternity in multiparas as a predominant high risk factor for preeclampsia over prolonged birth intervals: A study of 33,000 singleton pregnancies in Reunion Island
Source: PLoS One. 2024 Dec 23;19(12):e0312507. doi: 10.1371/journal.pone.0312507 (PMC11665996; doi:10.1371/journal.pone.0312507)
Supplement: S1 Checklist — (DOCX) [file pone.0312507.s001.docx]

- Formatting. **I think that the format is well now**- English language editing including grammar checks. **Checked by Professor Dekker**- The graphs should not be in 3D and must conform to the graphing standards of PloS One.

**The graph is in 2 D**
- The tables must be formatted to the requirements of PloS One.

**I think they are now**

- A graphical abstract is recommended but not compulsory.

Kind Regards

JOURNAL REQUIREMENTS:

1. Please ensure that the author list and affiliations are correct on the title page of your manuscript, and that your author contributions, competing interests, and financial disclosure are correct as listed below. All of these sections will be indexed in PubMed and published by PLOS ONE as you have written them. Please email plosone@plos.org if any changes to this content need to be made.
 **ALL ASSESMENTS ARE CORRECT FOR ALL CO-AUTHORS**Pierre-Yves Robillard:
Conceptualization
Data curation
Formal analysis
Investigation
Methodology
Supervision
Validation
Writing – original draft
Writing – review & editing

Silvia Iacobelli:
Funding acquisition
Resources
Supervision
Validation

Simon Lorrain:
Conceptualization
Formal analysis
Investigation
Methodology
Software
Validation

Francesco Bonsante:
Validation
Writing – review & editing

Malik Boukerrou:
Funding acquisition
Project administration
Resources
Validation

Marco Scioscia:
Supervision
Validation
Writing – review & editing

Phuong Lien Tran:
Data curation
Investigation

Gustaaf Dekker:
Conceptualization
Formal analysis
Methodology
Supervision
Writing – review & editing

Please see here for the full list and definition of contributor roles: <http://journals.plos.org/plosone/s/authorship#loc-author-contributions>

Please ensure that the Competing Interests and Financial Disclosure statements listed below are suitable for publication. These sections will be indexed in PubMed and published by PLOS ONE as you have written them. Please email plosone@plos.org if any changes to these statements need to be made.
**ALL ASSESMENTS ARE CORRECT competing interests and financial disclosure**
Competing Interests:
The authors have declared that no competing interests exist.

Financial Disclosure:
The author(s) received no specific funding for this work.

2. "The number of Supporting Information captions in your manuscript does not match the number of Supporting Information files uploaded to Editorial Manager. Please ensure that the Supporting Information files you have uploaded match the number of captions in your Supporting Information Captions section.
A Supporting Information file containing multiple supporting figures, tables, etc. is acceptable; however, it must have just one caption in the Supporting Information Captions section of the manuscript.
Please see these examples for more guidance: [http://journals.plos.org/plosone/s/file?id=wjVg/PLOSOne_formatting_sample_main_body.pdf"](http://journals.plos.org/plosone/s/file?id=wjVg/PLOSOne_formatting_sample_main_body.pdf%22)

3. To prevent production delays, we recommend using the Author Formatting Checklist to confirm that your paper meets PLOS ONE's typesetting requirements for References, Tables, and Figures: <http://journals.plos.org/plosone/s/file?id=c819/plos-one-author-formatting-checklist.docx>.

This checklist is a reference tool for you; please do not upload the completed Author Formatting Checklist with your submission files.

**VERIFIED, ALL POINTS OF THE CHECK LIST ARE GOOD**
4. To ensure your figures meet our technical requirements, please run each figure included in your submission files through the PACE tool: <https://pacev2.apexcovantage.com/>. PACE will assess whether your figures meet our technical requirements and will fix the figure(s) or identify any problem(s) that cannot be automatically fixed. It can also convert figures to TIFF format, resize, and rename figures to meet our naming conventions.
To use PACE, first register as a user. Follow the instructions on the site for assessing and converting your figure files. If you experience any difficulty using this tool or have questions about any of the figures and/or images in your paper, please inform the journal office in your response letter.
 **I had problems to register and use PACE, sorry**CONFIDENTIAL: This email and any attachments are confidential and for the sole use of the individual(s) to whom they are addressed. If you have received this message in error please delete the message and notify plosone@plos.org.

__________________________________________________
In compliance with data protection regulations, you may request that we remove your personal registration details at any time.  (Use the following URL: <https://www.editorialmanager.com/pone/login.asp?a=r)>. Please contact the publication office if you have any questions.
