# Supplementary material for: Primipaternity in multiparas as a predominant high risk factor for preeclampsia over prolonged birth intervals: A study of 33,000 singleton pregnancies in Reunion Island
Source: PLoS One. 2024 Dec 23;19(12):e0312507. doi: 10.1371/journal.pone.0312507 (PMC11665996; doi:10.1371/journal.pone.0312507)
Supplement: Attachment — Submitted filename: plos one paper review comments.docx [file pone.0312507.s003.docx]

**Overall comments:**

The manuscripts need major proof reading and revision for English grammar and syntax- recommend the use of proofreader and editor.

Manuscript to be justified for alignment.

Paper title very similar to:

Primipaternity and birth interval; independent risk factors for preeclampsia

[Primipaternity and birth interval; independent risk factors for preeclampsia: The Journal of Maternal-Fetal & Neonatal Medicine: Vol 33 , No 2 - Get Access (tandfonline.com)](https://www.tandfonline.com/doi/full/10.1080/14767058.2018.1489794)

*Positives:*

- Very detailed analysis and interpretation of results
- Novel study in term of cohort and factors studied.
- Large sample size provides more realistic picture of the risk factors
- Wealth of data to write other papers
- Study period of 24 years also gives credibility to the results showing changes over time

*Negatives:*

- Authors do not mention the specificity of the cohort having any bearing on the results. Other studies use different populations hence the disparity between results, different cultural and social and genetic backgrounds will yield different results.
- Study lacks theories or hypothesis as to why the results are such but rather authors explain the findings without explanation.
- Different font sizes and types show different sections written by different people but not carefully synthesized into one coherent paper.

Specific areas of improvement:

Title

1. spacing after colon
2. 33.000 is different to 33 000 or 33,000- please rectify. Also population was in excess of 33 000

Abstract

3. Different font types found in abstract subheadings
4. Design: incomplete sentence: …at South-Reunion University’s maternity ?????? (Reunion Island, Indian Ocean) over 24 years….
5. Design needs to be more comprehensive.
6. Full stop after sub head “main outcome measures” to be removed
7. The results section is very unstructured and untidy.
8. Conclusion – word intervals the “I” is bolded

Introduction

9. References are not consistent i.e. full stop after et al
10. Use of brackets after reference number [8,9] missing a closed bracket

MATERIAL AND METHODS.

11. Word missing...”at the maternity ???” ward, department or unit?
12. Elaborate on how gestational hypertension and eclampsia were defined?
13. paragraph 5 after **“Definition of exposure and outcomes”: ore-pregnancy should read pre-pregnancy**

Results:

14. results need to be neatened up; data is all over the place.
15. Narrative on page 15 for figure has duplication of % sign
16. Check all figures containing decimals sone are represented as < 0..0001

Discussion

17. Needs major proofreading and editing
18. Discussion is very results and statistics heavy it needs more theory and explanation of the results
