# Supplementary material for: Primipaternity in multiparas as a predominant high risk factor for preeclampsia over prolonged birth intervals: A study of 33,000 singleton pregnancies in Reunion Island
Source: PLoS One. 2024 Dec 23;19(12):e0312507. doi: 10.1371/journal.pone.0312507 (PMC11665996; doi:10.1371/journal.pone.0312507)
Supplement: Attachment — Submitted filename: Critics reviewers.docx [file pone.0312507.s004.docx]

**REVIEWERS’ COMMENTS**.

**1. EDITOR COMMENTS. Answers in green in the text**

1. Please ensure that your manuscript meets PLOS ONE's style requirements, including those for file naming. The PLOS ONE style templates can be found at 
https://journals.plos.org/plosone/s/file?id=wjVg/PLOSOne_formatting_sample_main_body.pdf and 
https://journals.plos.org/plosone/s/file?id=ba62/PLOSOne_formatting_sample_title_authors_affiliations.pdf

2. Please include your full ethics statement in the ‘Methods’ section of your manuscript file. In your statement, please include the full name of the IRB or ethics committee who approved or waived your study, as well as whether or not you obtained informed written or verbal consent. If consent was waived for your study, please include this information in your statement as well.

We have added, page 6 : **Ethics approval: This study was conducted in accordance with French legislation. As per new French law applicable to trials involving human subjects (Jardé  Act), a specific approval of an ethics committee (comité de protection des personnes- CPP) is not required for this non-interventional study based on retrospective, anonymized data of authorized collections and written patient consent is not needed. Patients and Public involvement. The South-Reunion perinatal database (since 2001) includes 264 items. It is considered as a fully medical database , datasheets are electronically completed solely by midwives, obstetricians and neonatologists. All epidemiological studies are obligatorily performed on anonymized data (French law). As such, there is no direct patient or public involvement.**

Additional Editor Comments:

The following editorial comments must be addressed:

- Please revise the manuscript to meet the required formatting guidelines presented on our website. <https://journals.plos.org/plosone/s/submission-guidelines>

- The substance of the work is noted however the authors should refrain from discrediting other authors but rather scientifically contrast the different viewpoints of the argument. The entire manuscript needs to be rewritten to remove this as it presents an ethical non-conformance for PloS One. These types of comments are mainly evident in the introduction and conclusion of the manuscript. One example is as follows: (“The 2002 Skjaerven et al.'s statement: After adjustment for the interval birth, a change of partner is not associated with an increased risk of preeclampsia” severely disorientated the preeclampsia debate in a wrong direction during two decades.).

**We have re-written the INTRODUCTION, deleting 6 lines (essentially after new modification now in dark gree in the text). The modifications beginning at the 3rd paragraph. We have deleted the sentence « was published in the very prestigious NEJM ».**

**The new text now is :**

« The Skjaerven et al.’s **NEJM paper** had a tremendous impact **as it directly disagreed** with our preceding publications on the importance of ‘primipaternity’ in 1993 and 1994 (the last one in The Lancet) [4,5]. In those studies we showed that in Guadeloupe (French West Indies) a change of paternity for the index pregnancy, based on direct inquiries with multiparas, was strongly associated with PE [5]. **Over the past decades the relative importance of prolonged birth interval versus primipaternity has remained a controversial topic**.. From an etiological perspective, **this ongoing scientific debate** is not a trivial one; evidently, the primipaternity paradigm is in line with the fundamental concept that human **placentation** may be considered as a “fetal hemigraft” , and as such the classic superficial cytotrophoblast invasion **of the spiral particularly in early-onset PE with fetal growth restriction** could therefore represent a type of immunological maladaptation of this fetal hemigraft [7-10]. On the other hand, prolonged interval between pregnancies in multiparas as a direct major risk factor for PE appears to be more in line with a kind of vascular maternal problem that increases progressively with time (a kind of “aging approach”). This was also supported by Tanberg et al. (also in Norway) who concluded on a cohort of 500,000 mothers after assisted reproductive technologies (ART, period 1988 to 2009 – so with a ten year overlap of the prior Skjaerven cohort) that the PE risk may increase by parity, interbirth interval and advanced maternal age, but with not with change of father or smoking [11]. In contrast, in 2000 a study published by Li and Wi based on 140,147 women with two consecutive births (Californian birth certificate 1989-1991) [12] among women without PE/eclampsia in the 1st pregnancy, changing partners resulted in a 30% increase in the risk in the subsequent pregnancy compared with those who did not change partner (95% CI: 1.1-1.6). On the other hand, among women with PE in the 1st birth, changing partner resulted in a 30% reduction in the risk of PE in the subsequent pregnancy (95% CI: 0.4- 1.2). Interbirth interval was very unlikely to be a confounder in the Li and Wi study since the authors restricted their population to births that were between 1-3 years apart [12]. Hercus and Dekker in an Australian population studied this problem in 2020 [13] and concluded that “both prolonged birth intervals and primipaternity are independent risk factors for preeclampsia in multigravidae”.

**Notwithstanding the fact that we have previously discussed some concerns regarding the Skjaerven’s study [6-8], it is clear that the relative importance of prolonged birth interval versus primipaternity as PE risk factor in multiparous women still represents an important fundamental research question.**

The aim of this study was to address this fundamental question **by a comprehensive analysis of our 23year** pregnancy cohort in Reunion island using **a detailed high quality perinatal database** where we have an item “changing paternity” by direct inquiry to the women (PE and controls).”

**IN THE CONCLUSION we deleted the sentence**

**“The 2002 Skjaerven et al.'s  statement: “After adjustment for the interval birth, a change of partner is not associated with an increased risk of preeclampsia” severely disorientated  the preeclampsia debate in a wrong direction during two decades. »**

- The rationale and aim of the study must be rewritten with clarity and in an acceptable scientific format.

**In the abstract, we modified :**

**Objectives : To evaluate the relative importance of changing paternity (“primipaternity”) in multiparas versus prolonged birth/pregnancy interval as risk factors for preeclampsia (PE).**

**Main outcome Measures**. **The aim of this study was to analyze the problem in our population using our detailed perinatal database where we have an item “changing paternity” by direct inquiry to the women (preeclamptic and controls). Comparison of risk factors for PE between multiparae with a new male partner for the index pregnancy versus stable couples.**

- The materials and methods must be written logically and scientifically in alignment with the work and claims made. Segmenting the methods will help in creating scientific flow which will enhance the readability. Importantly the study population size and design is not clear.

**At the beginning of material and methods we added:**

**The aim of this study was to evaluate the relative importance of changing paternity (“primipaternity”) in multiparas versus prolonged birth/pregnancy interval as risk factors for preeclampsia (PE) using in our perinatal database the item “changing paternity” by direct inquiry to the women (preeclamptic and controls). Comparison of risk factors for PE between multiparae with a new male partner for the index pregnancy versus stable couples.**

Page 5, we wrote :

**Definition of exposure and outcomes *.* During the 23-year period all consecutive singleton pregnancies after 22 weeks gestation have been analysed. For multiparas, women who had changed the male partner for the index pregnancy were considered as cases, those who did not were the controls.**

- The manuscript must be edited for English, neatness, syntax, grammar and ensure that it is written scientifically.

**Professor Dekker verified entierly the text**

- **The title must be revised to be more concise** without being similar to other articles already published and aligned to the actual work done.

THE NEW TITLE IS NOW :

**PRIMIPATERNITY IN MULTIPARAS IS HIGHLY PREDOMINANT AS RISK FACTOR FOR PREECLAMPSIA OVER PROLONGED BIRTH INTERVALS. A STUDY OF 33.000 SINGLETON PREGNANCIES IN REUNION ISLAND**

SHORT TITLE :

**PREECLAMPSIA AND MULIPARAS: PRIMIPATERNITY IS A HIGHER RISK FACTOR THAN PROLONGED BIRTH INTERVALS**

- It is suggested that the authors use statistical graphs where possible to clearly represent the results.

**We feel that Figure 2 : Significant adjusted odds ratios of several risk factors for early (EOP) and late onset (LOP) preeclampsia.**

**Although unusual gives a visual direct impact for the real impact of the 2 risk factors studied (all results adjusted Odds-ratios)**

- The results section is inconsistent and needs to be rewritten. The results must be clearly specified which must be in alignment with the title and entire rationale of the study.

**We have made modifications (dark green in the text)**

- The discussion must be revised for coherency and to critically discuss the key findings of the presented study.

**We have modified a lot the discussion (through the 2 reviewers’ suggestion). We came also from 14 references to 23**

- The authors should include a conflict of interest and discloser statement as per the Plos ethical guidelines and manuscript template

At the end of Material and methods we added : « **Conflict of interest. The authors report no conflict of interest ».**

The following reviewer comments must be addressed:

**2. Reviewer 1. Answers in red in the text**

Overall comments:

Positive Comments:
• Very detailed analysis and interpretation of results
• Novel study in term of cohort and factors studied.
• Large sample size provides more realistic picture of the risk factors
• Wealth of data to write other papers
• Study period of 24 years also gives credibility to the results showing changes over time
Areas to Address:

• The authors do not mention the specificity of the cohort having any bearing on the results. Other studies use different populations hence the disparity between results, different cultural and social and genetic backgrounds will yield different results.

We have added in strength and limitation, page 14 : « **The specificity of our cohort with cultural and social and genetic backgrounds different than other already published studies made in different populations could yield different results. Reunion Island is a French department in the Southern Indian Ocean. The peculiarity of this tropical region lays in the multiethnic origin of inhabitants: Africa and intermixed population (50%), Europe (27%), India (20%) and China (3%). However, we feel it can be a strength for this particular study: this territory witnessed two centuries of slavery until 1848, where official marriages were forbidden for slaves by masters. These communities then reacted by reproduction being often with different fathers in successive pregnancies. This scheme remains embedded in this society, where it is not a problem for women to say if a father is a new one or not (well-known pattern which has been described for 4-5 decades by demographers as “Women Family Structures” in the Carribbeans or in American areas where slavery existed).** “

• Study lacks theories or hypothesis as to why the results are such but rather authors explain the findings without explanation.

We have added, page 14 a long paragraph explaining the biological plausibility of the « primipaternity concept » : « **What is the biologic explanation for the primipaternity paradigm ? To date, two major hypotheses (which may be complementary) and are extensively developed in some recent paper [8, 16]: First, David Haig’s paternal-maternal conflict in every pregnancy [17], second, the immunological one [18,19] also associated with the concept of a necessary long sperm exposure (paternal tissues) in first pregnancies results in a partner specific mucosal tolerance [20]. Immunology of reproduction has made giant leaps during the last two decades: The haemochorial placenta in primates, and in particular in humans with the deepest invasion represents a scenario where the mother is facing a more or less human-specific major immune challenge, the “fetal hemi-allograft paradox ” [19] first mentioned by Medawar, Prior pregnancies in the same relationship translate in developped ta “trained immunological memory” in line with well known epidemiologic findings of lower rates o f of PE , fetal growth restriction, fetal demise, and low birthweight in subsequent pregnancies. Also, we now appreciate that shallow endovascular trophoblast invasion is primarily linked to IUGR (with or without the maternal syndrome of preeclampsia) [22, 23].**

• Different font sizes and types show different sections written by different people but not carefully synthesized into one coherent paper. **Corrected**

Other specific comments per section:


Introduction
1. References are not consistent i.e. full stop after et al. **This reference has been deleted (critic on self-citation)**

2. Use of brackets after reference number [8,9] missing a closed bracket

corrected

MATERIAL AND METHODS.
3. Word missing...”at the maternity …” ward, department or unit?

Corrected : department
4. Elaborate on how gestational hypertension and eclampsia were defined?

We have modified page 5 : **”Preeclampsia was defined according to the World Health Organization recommendations [14] and the International Society for the study of Hypertension in Pregnancy ) relatively to the guidelines in force at the year of pregnancy. [15] as the new onset of hypertension (BP ≥140 mmHg systolic or ≥90 mm Hg diastolic) at or after 20 weeks’ gestation and substantial proteinuria (> 0.3 g/24 hours). Early onset preeclampsia was defined as preeclampsia that developed before 34 weeks of gestation.”**

5. paragraph 5 after “Definition of exposure and outcomes”: ore-pregnancy should read pre-pregnancy. Corrected

Results:
6. results need to be neatened up; data is scattered. We divided the results to be connected with the different Tables as it is recommended by PLOS (Tables embeded inside the text)

7. The narrative on page 15 for the figure has a duplication of the % sign. **Corrected thank you**
8. Check all figures containing decimals sone are represented as < 0..0001. **Corrected thank you**

9 Discussion. Discussion is very results and statistics-heavy heavy it needs more theory and explanation of the results. **Paragraph added page 14, pleas see above**

10. The authors stated “All relevant data are within the manuscript and its Supporting Information files”, but there is no additional data file available.

**Reviewer 2. Answers in blue in the text**

Comment #1: In the Abstract, Design and Main Outcome Measures should be restructured and described in more details related to PE risk factors, not only stated “… on obstetrical and neonatal risk factors” (?).
We have precised : « **obstetrical and neonatal risk factors (156 items in total, of which, chronic or gestational hypertension, proteinuria, HELLP syndrome).”**

Comment #2: The objective of this study should be rephrased, clearly define the “problem” and without parentheses: “The aim of this study was to analyze the problem in our 24-year cohort in Reunion island using our detailed perinatal database where we have an item “changing paternity” by direct inquiry to the women (preeclamptic and controls).

The sentence is now :  **»The aim of this study was to analyze the problem in our using our detailed perinatal database where we have an item “changing paternity” by direct inquiry to the women (preeclamptic and controls). Comparison of risk factors for PE between multiparae with a new male partner for the index pregnancy versus stable couples.”**

Comment #3: The study period “from January 1st, 2001, to December 31, 2023” is 23 years. **Corrected thank you**

Comment #4: In Results, tables and figures should be clearly formatted and figures with better resolution.

Comment #5: The authors stated “All relevant data are within the manuscript and its Supporting Information files”, but there is no additional data file available.

Comment #6: Excessive self-citation should be avoided and referencing the works from others are encouraged.

**The corrected paper has now 23 references (instead of 14, original paper) with different authors. W have deleted the former ref 14 (Robillard et al)**

Comment #7: The manuscript should be checked carefully by a native for English spelling and grammar.

Comment #8 : the niche population which could assist in explaining the results are not mentioned. e.g. is it a developing country with poor healthcare standards. lack of education hence seeking multiple fathers etc.
 In pages 15 and 16, we devopped :

The strength of our study is, first, the capturing of all perinatal outcomes in our maternity, European standard of care. With 4,300 births per year, the university maternity, level 3, represents 82% of all births in the south of the island. but, as level 3 manages all cases of PE .The data in this large cohort are homogeneous as they were collected in a single center (lower variability) and not based on national birth registers but directly from medical records (avoiding inadequate codes**). Second: Reunion island is an overseas department of France in the Indan ocean, and , as such, has the highest per capita gross domestic income of the area (e.g South-Africa, Mozambique, Madagascar, Mauritius island, Seychelles etc….). Further, access to maternity care free of charge as provided by the French healthcare system (nationwide French “social security” system) which combines freedom of medical practice with social security. Hospitals have European standards of care and pregnant have an average of 8 prenatal visits and 4 ultrasounds during their pregnancy .Third,** **The specificity of our cohort with cultural and social and genetic backgrounds different than other already published studies made in different populations could yield different results. Reunion Island is a French department in the Southern Indian Ocean. The peculiarity of this tropical region lays in the multiethnic origin of inhabitants: Africa and intermixed population (50%), Europe (27%), India (20%) and China (3%). However, we feel it can be a strength for this particular study: this territory witnessed two centuries of slavery until 1848, where official marriages were forbidden for slaves by masters. These communities then reacted by reproduction being often with different fathers in successive pregnancies. This scheme remains embedded in this society, where it is not a problem for women to say if a father is a new one or not (well-known pattern which has been described for 4-5 decades by demographers as “Women Family Structures” in the Caribbean’s or in American areas where slavery existed).**

3. Have the authors made all data underlying the findings in their manuscript fully available?

**Results added in supplementary file**
