# Supplementary material for: Primipaternity in multiparas as a predominant high risk factor for preeclampsia over prolonged birth intervals: A study of 33,000 singleton pregnancies in Reunion Island
Source: PLoS One. 2024 Dec 23;19(12):e0312507. doi: 10.1371/journal.pone.0312507 (PMC11665996; doi:10.1371/journal.pone.0312507)
Supplement: Attachment — Submitted filename: CRITICS REVISION 2 Plos one.docx [file pone.0312507.s005.docx]

1. **Journal requirements**

Please review your reference list to ensure that it is complete and correct. If you have cited papers that have been retracted, please include the rationale for doing so in the manuscript text, or remove these references and replace them with relevant current references. Any changes to the reference list should be mentioned in the rebuttal letter that accompanies your revised manuscript. If you need to cite a retracted article, indicate the article’s retracted status in the References list and also include a citation and full reference for the retraction notice.

**We have verified the references which are in the format such as :**

**Kho EM, McCowan LM, North RA, Roberts CT, Chan E, Black MA, Taylor RS, Dekker GA; SCOPE Consortium. Duration of sexual relationship and its effect on preeclampsia and small for gestational age perinatal outcome. J Reprod Immunol. 2009 Oct;82(1):66-73. doi: 10.1016/j.jri.2009.04.011. Epub 2009 Aug 12. PMID: 19679359.**

**2. Editorial Comments: ANSWER IN DARK GREEN IN THE TEXT**

Additional Editor Comments:

Thank you for your revisions. We however believe that there are some minor revisions that need to be made before we consider your manuscript for publication. They are as follows:

**Thank you for these very postive critics which improve the quality f the text. WE HAVE REMOVED FIGURE 1 WHICH ALSO AS YOU SAY** « are okay for power point presentations but not for publication purposes. ». Therefore, former Figure 2 becomes Figure 1

General:
- Inclusion of more scientific information in the methods section as indicated.
- Revision of statements for consistency and coherency
- Correct English and grammar
- Correct statements to a more scientific format
-Check refencing consistency according to PlOs One policies


Title suggestion:

Primipaternity in Multiparas as a Predominantly High Risk Factor for Preeclampsia Over prolonged birth intervals: A study of Singleton Pregnancies in Reunion Island

**The new title is now (as also sggested by reviewer 1) is :**

**PRIMIPATERNITY IN MULTIPARAS AS A PREDOMINANT HIGH RISK FACTOR FOR PREECLAMPSIA OVER PROLONGED BIRTH INTERVALS : A STUDY OF 33,000 SINGLETON PREGNANCIES IN REUNION ISLAND**

Abstract:
Segment Abstract according to the following sections

- Introduction
- Methods – Mention briefly the actual methods used. There is mention of assessment but not what the actual assessment is about.

**We have modified :**

**Objectives** : To evaluate the relative importance of changing paternity (“primipaternity”, **direct inquiry with patients**) in multiparas versus prolonged birth/pregnancy interval as risk factors for preeclampsia (PE) **by a logistic regression model comparing the adjusted odds ratios of both exposures.**

- Results
- Conclusion and Relevance
**Conclusions. Primipaternities in multiparae belongs to the major risk factors such as history of preeclampsia, chronic hypertension, multiple pregnancies while prolonged birth intervals belongs to moderate “physiological or aging influence” such as increasing maternal age, parity or increasing pre-pregnancy BMI.**

Introduction:
I recommend that the authors change the following paragraphs:

Interestingly Trogstadt et al (with Skjaerven as one of the co-authors) looked at the effect of prior miscarriage/abortions (< 22 week’s gestation) in nulliparous women (Norwegian MoBa cohort) [3] and showed that prior abortion with the same partner reduced the risk for PE but not in women with new paternity pregnancies and the authors concluded that normal pregnancies interrupted at early gestation may induce immunological changes that reduce the risk of preeclampsia in a subsequent pregnancy.
The Skjaerven et al.’s NEJM paper had a tremendous impact as it directly disagreed with our preceding publications on the importance of ‘primipaternity’ in 1993 and 1994 (the last one in The Lancet) [4,5].

To:
Interestingly, Trogstadt et al. investigated the effect of prior miscarriage/abortions (< 22 week’s gestation) in nulliparous women within a Norwegian MoBa cohort [3]. This study identified that prior to abortion with the same partner a reduced risk of PE was observed however, not in women with new paternity pregnancies. These findings suggested that normal pregnancies interrupted at early gestation may induce immunological changes that reduce the risk of preeclampsia in subsequent pregnancies.
Importantly, a study by Skjaerven et al.’s [add reference] had tremendous impact as it directly contrasted our preceding publications on the importance of ‘primipaternity’ [4,5].

**NEW TEXTMODIFIED** : **Interestingly, Trogstadt et al. investigated the effect of prior miscarriage/abortions (< 22 week’s gestation) in nulliparous women within a Norwegian MoBa cohort [3]. This study identified that prior to abortion with the same partner a reduced risk of PE was observed however, not in women with new paternity pregnancies. These findings suggested that normal pregnancies interrupted at early gestation may induce immunological changes that reduce the risk of preeclampsia in subsequent pregnancies. Importantly, a study by Skjaerven et al.’s [1] had tremendous impact as it directly contrasted our preceding publications on the importance of ‘primipaternity’ [4,5].**


Methods:

Add more details on the following methods and parameters applied per the software package used since multiple software platforms were used:
‘Epidemiological data have been recorded and analysed with the software EPI-INFO 7.1.5 (2008, CDC Atlanta, OMS), EPIDATA 3.0 and EPIDATA Analysis V2.2.2.183. Denmar’

We have added : .**EPIDATA allowing the adaptation for WINDOWS 10 of the former EPI-INFO (MS DOS) in complete cooperation with CDC Atlanta. All calculations were made then with and EPIDATA Analysis V2.2.2.183. Denmark.**

I suggest specifying the package used for each statistical test done and also adding more detail on the other tests done using the software.
**All calculations were made then with and EPIDATA Analysis V2.2.2.183. Denmark.**

Results:

- Intext references to tables and figures should be included and must be consistent. **DONE**

- Authors must adjust figure to a 2D format the 3D format is not for scientific publication as a publication must be as clear as possible to read.

**FIGURE 1 (FORMER FIGURE 2) IS NOW IN 2D FORMAT**

- Authors must not use bullets to disseminate their findings and must write their findings in a clear scientific manner for coherency, the bullet points are okay for power point presentations but not for publication purposes.

**WE HAVE DELETED THE BULLETS AS WELL IN THE RESULTS AS IN THE DISCUSSION**

Discussion
- Authors must not use bullets to discuss their work and must write their findings in a clear scientific manner for coherency, the bullet points are okay for power point presentations but not for publication purposes.

**WE HAVE DELETED THE BULLETS AS WELL IN THE RESULTS AS IN THE DISCUSSION**

- I recommend the authors change the following paragraph:
What is the biologic explanation for the primipaternity paradigm ? To date, two major hypotheses (which may be complementary) and are extensively developed in some recent paper [8, 16]: First, David Haig’s paternal maternal conflict in every pregnancy [17], second, the immunological one [18,19] also associated with the concept of a necessary long sperm exposure (paternal tissues) in first pregnancies results in a partner specific mucosal tolerance [20]. Immunology of reproduction has made giant leaps during the last two decades: The haemochorial placenta in primates, and in particular in humans with the deepest invasion represents a scenario where the mother is facing a more or less human-specific major immune challenge, the “fetal hemi-allograft paradox ” [19] first mentioned by Medawar, Prior pregnancies in the same relationship translate in developped ta “trained immunological memory” in line with well known epidemiologic findings of lower rates o f of PE , fetal growth restriction, fetal demise, and low birthweight in subsequent pregnancies. Also, we now appreciate that shallow endovascular trophoblast invasion is primarily linked to IUGR (with or without the maternal syndrome of preeclampsia) [22, 23]

TO

In explaining the the primipaternity paradigm, to date, two major hypotheses (which may be complementary) and are extensively developed in some recent paper [8, 16]. Firstly, David Haig’s (check refencing) paternal maternal conflict in pregnancy [17] and secondly, the immunological challenge [18,19] which is also associated with the concept of a necessary long sperm exposure (paternal tissues) in first pregnancies results in a partner specific mucosal tolerance [20]. The haemochorial placenta in primates, and in particular in humans with the deepest invasion represents a scenario where the mother is facing a more or less human-specific major immune challenge, the “fetal hemi-allograft paradox ” [19] first mentioned by Medawar, prior pregnancies in the same relationship translate in developed “trained immunological memory” in line with well-known epidemiologic findings of lower rates of PE , fetal growth restriction, fetal demise, and low birthweight in subsequent pregnancies. Moreover, we now appreciate that shallow endovascular trophoblast invasion is primarily linked to IUGR (with or without the maternal syndrome of preeclampsia) [22, 23]
 **THE NEW PARAGRAPH IS NOW :**

**In explaining the the primipaternity paradigm, to date, two major hypotheses (which may be complementary) and are extensively developed in some recent paper [8, 16]. Firstly, David Haig’s (check refencing) paternal maternal conflict in pregnancy [17] and secondly, the immunological challenge [18,19] which is also associated with the concept of a necessary long sperm exposure (paternal tissues) in first pregnancies results in a partner specific mucosal tolerance [20]. The haemochorial placenta in primates, and in particular in humans with the deepest invasion represents a scenario where the mother is facing a more or less human-specific major immune challenge, the “fetal hemi-allograft paradox ” [19] first mentioned by Medawar, prior pregnancies in the same relationship translate in developed “trained immunological memory” in line with well-known epidemiologic findings of lower rates of PE , fetal growth restriction, fetal demise, and low birthweight in subsequent pregnancies. Moreover, we now appreciate that shallow endovascular trophoblast invasion is primarily linked to IUGR (with or without the maternal syndrome of preeclampsia) [22, 23]**

- Remove etc from the following sentence and change further to furthermore: (e.g South-Africa, Mozambique, Madagascar, Mauritius island, Seychelles etc….). Further,

**DONE: Furthermore**


**3. Reviewer Recommendations: ANSWERS IN RED IN THE TEXT**

Comment #1: At the end of Materials & methods, there is the section “Fundings: The author(s) received no specific funding for this work. Conflict of interest. The authors report no conflict of interest”, this should be removed and inserted at the appropriate position.

**These have been put in final disclosure**

Comment #2: In Results, tables and figures should be clearly formatted and figures with better resolution

Comment #3: Many punctuation errors should be corrected. **Done**


[Note: HTML markup is below. Please do not edit.]

Reviewers' comments:

Reviewer's Responses to Questions

**Comments to the Author**

1. If the authors have adequately addressed your comments raised in a previous round of review and you feel that this manuscript is now acceptable for publication, you may indicate that here to bypass the “Comments to the Author” section, enter your conflict of interest statement in the “Confidential to Editor” section, and submit your "Accept" recommendation.

Reviewer #1: All comments have been addressed

2. Is the manuscript technically sound, and do the data support the conclusions?

The manuscript must describe a technically sound piece of scientific research with data that supports the conclusions. Experiments must have been conducted rigorously, with appropriate controls, replication, and sample sizes. The conclusions must be drawn appropriately based on the data presented.

Reviewer #1: Yes

3. Has the statistical analysis been performed appropriately and rigorously?

Reviewer #1: Yes

4. Have the authors made all data underlying the findings in their manuscript fully available?

Reviewer #1: Yes

5. Is the manuscript presented in an intelligible fashion and written in standard English?

PLOS ONE does not copyedit accepted manuscripts, so the language in submitted articles must be clear, correct, and unambiguous. Any typographical or grammatical errors should be corrected at revision, so please note any specific errors here.

Reviewer #1: No **Professor Dekker revised the English language**

6. Review Comments to the Author

Please use the space provided to explain your answers to the questions above. You may also include additional comments for the author, including concerns about dual publication, research ethics, or publication ethics. (Please upload your review as an attachment if it exceeds 20,000 characters)

Reviewer #1: PONE-D-24-23454R1
RELATIVE INFLUENCE OF PRIMIPIPATERNITY AND PROLONGED BIRTH INTERVAL INMULTIPARAE AS INDEPENDENT RISK FACTORS FOR PREECLAMPSIA;A STUDY OF 33.000 SINGLETON PREGNANCIES IN REUNION ISLAND
changed into
PRIMIPATERNITY IN MULTIPARAS IS HIGHLY PREDOMINANT AS RISK FACTOR FOR PREECLAMPSIA OVER PROLONGED BIRTH INTERVALS. A STUDY OF 33.000 SINGLETON PREGNANCIES IN REUNION ISLAND

**The new title (as suggested also by the Editor) is :**

**PRIMIPATERNITY IN MULTIPARAS AS A PREDOMINANT HIGH RISK FACTOR FOR PREECLAMPSIA OVER PROLONGED BIRTH INTERVALS : A STUDY OF 33,000 SINGLETON PREGNANCIES IN REUNION ISLAND**

Most of comments and suggestions raised within the original manuscript have been solved in the revised version R1. The manuscript needs some minor revisions before being considered for publication.

Comment #1: At the end of Materials & methods, there is the section “Fundings: The author(s) received no specific funding for this work. Conflict of interest. The authors report no conflict of interest”, this should be removed and inserted at the appropriate position.

**These have been put in final disclosure**

Comment #2: In Results, tables and figures should be clearly formatted and figures with better resolution

**Figure 1 (former Figure 2) has been reformatted .**

**Former Figure 1 has been removed**
